# Supplementary material for: 3D collagen architecture induces a conserved migratory and transcriptional response linked to vasculogenic mimicry
Source: Nat Commun. 2017 Nov 21;8:1651. doi: 10.1038/s41467-017-01556-7 (PMC5698427; doi:10.1038/s41467-017-01556-7)
Supplement: Supplementary file 1 — Supplementary information [file 41467_2017_1556_MOESM1_ESM.pdf]

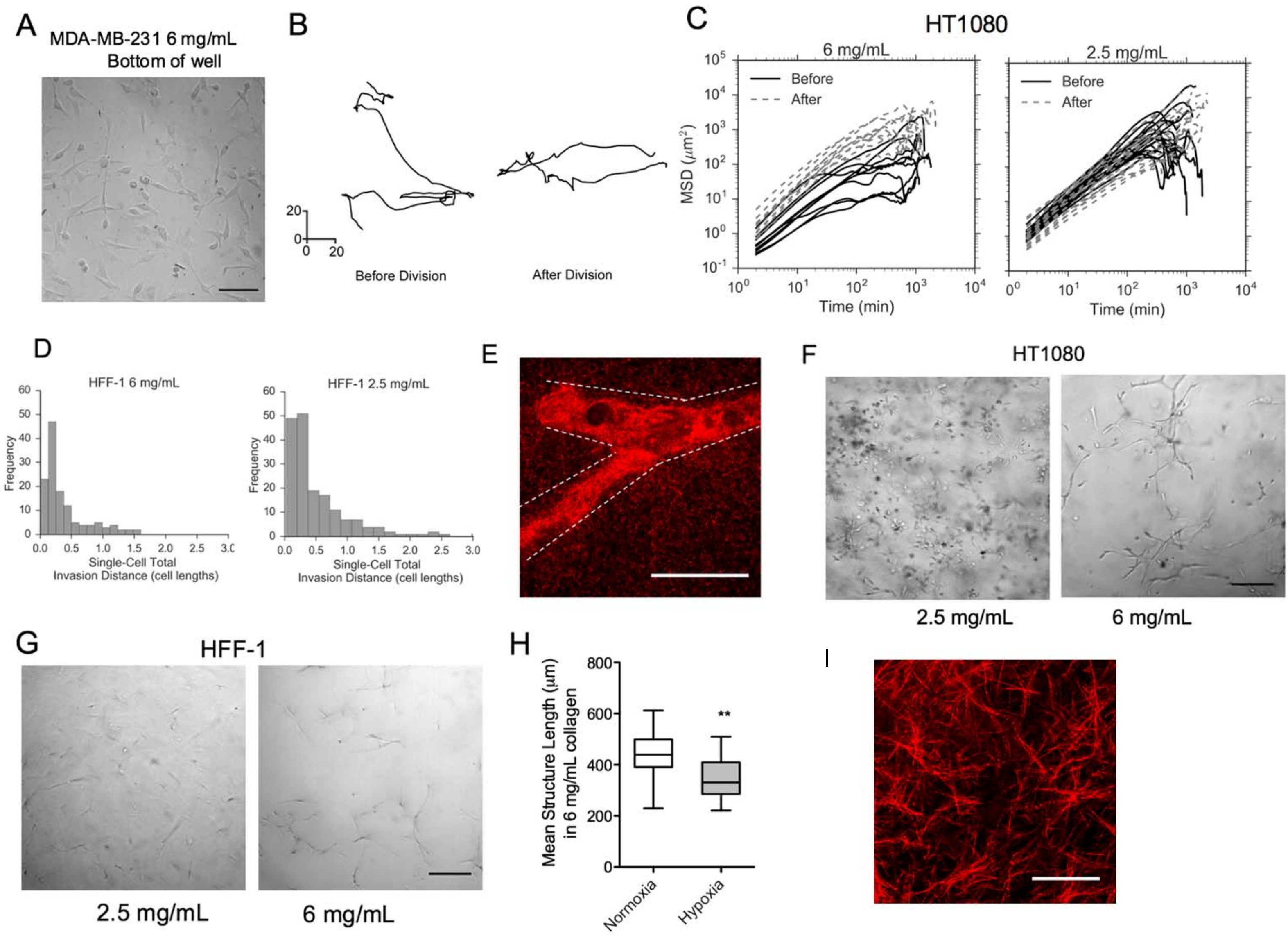

Supplementary Figure 1. A. Representative bright field image of MDA-MB-231 cells embedded in a 6mg/mL collagen gel but in contact with the coverslip. Scale bar 100 μm **B.** Representative trajectories of cells cells embedded in a 6mg/mL collagen gel but in close contact with the coverslip before and after cell division. The trajectories show no appreciable differences between the cell movement before or after division. **C.** Mean Squared Displacement (MSD) and persistent time of HT-1080 cells before and after cell division for cells in low density and high density collagen. MSDs shown are 12 representative cell trajectories. **D.** Total invasion distance of single cells and their progeny for HFF-1 fibroblasts cells in 6 mg/mL (left) and 2.5mg/mL (right) collagen gels in units of cell length (see methods) after 48 h of cell encapsulation. **E**. Representative confocal reflection image showing collagen fibers around a chain structure formed by MDA-MB-231 cells cultured in high density collagen gel for 7 days, dotted lines show the outline of the chain structure. Scale bar 100um. **F.** Representative bright field images of HT-1080 cells after 7 days of culture in 2.5 mg/mL (left) and 6 mg/mL (right) collagen I matrix. Scale bar 250 μm. **G.** Representative bright field images of HFF-1 fibroblast cells after 7 days of culture in 2.5 mg/mL (left) and 6 mg/mL (right) collagen I matrix. Scale bar 250 μm. **H.** Mean structure length formed by MDA-MB-231 cells cultured in high density 3D collagen after 7 days under normoxia (21% O_2) or hypoxia (1% O_2). Comparison was performed using Mann–Whitney U test. **I**. Representative confocal reflection image showing a 2.5mg/mL collagen gel polymerized at 20°C Scale bar 100 μm. Representative images of N=3 biological replicates for all experiments unless otherwise noted. Statistical significance is indicated as *, **, *** for p≤0.05, p≤0.01, p≤0001 respectively.

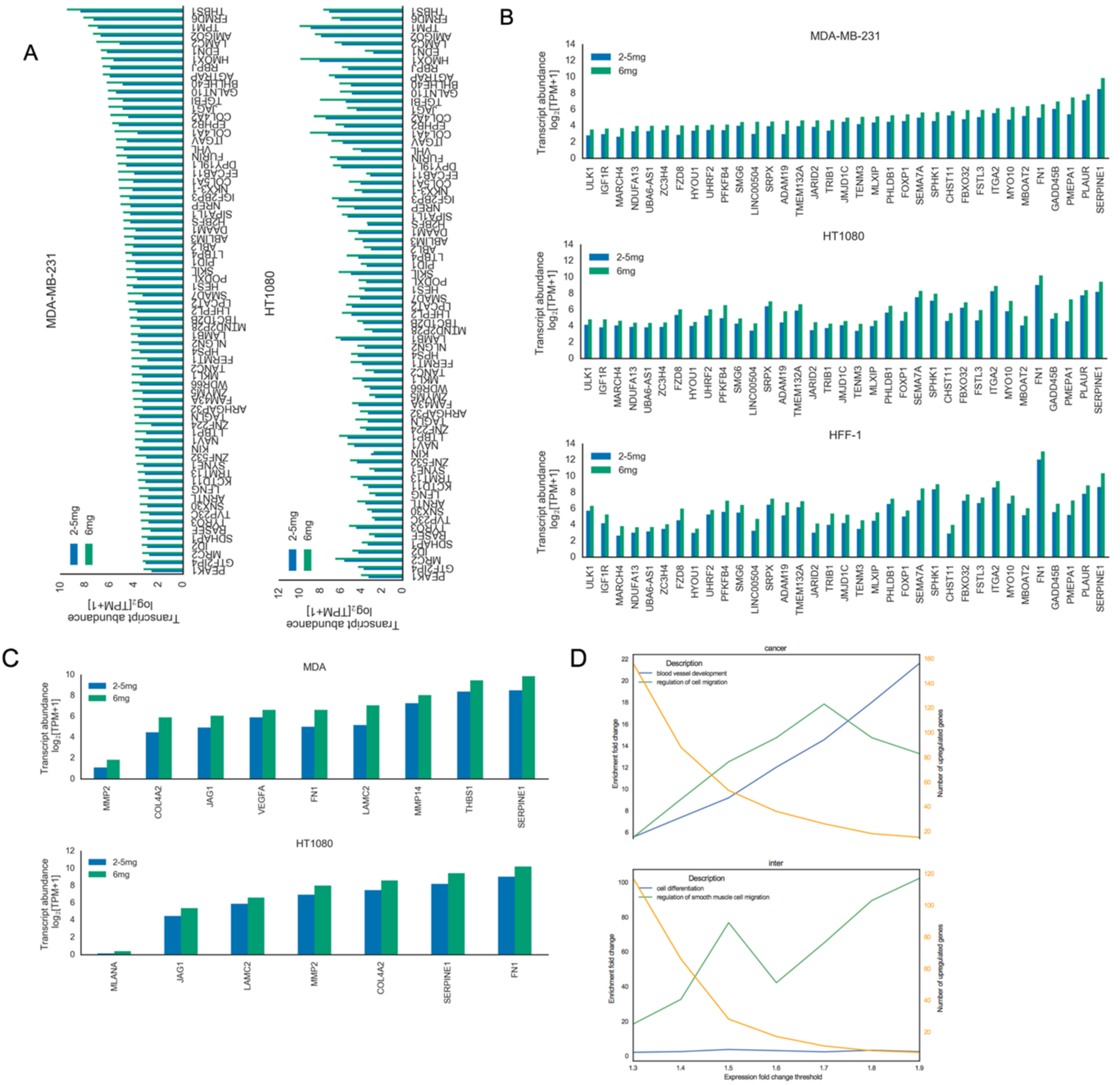

Supplementary Figure 2. A. Bar plot showing mean of n=3 expression values of the 70 genes upregulated by both cancer cell lines. MDA-MB-231 (top), genes sorted by low to high level of expression. HT1080 (bottom) gene order from top panel. **B.** Bar plot showing mean of n=3 expression values of the 35 genes upregulated by cancer cells and HFF-1 fibroblasts. MDA-MB-231 (top), genes sorted by low to high level of expression. HT1080 (middle) and HFF-1 (bottom) gene order from top panel **.C.** Mean of n=3 expression levels of genes previously reported as being involved in vasculogenic mimicry and upregulated by cancer cells in high density collagen. For this panel TPM>5 was not required for analysis. **D.** Sensitivity analysis of Gene Ontology Analysis presented in Figure 2. Left Panel: Plot showing number of genes included in the analysis as a function of fold change threshold (yellow) and fold enrichment of 2 key terms (blood vessel development and regulation of cell migration, blue and green respectively) for the two gene sets cancer specific (70 Genes) and common to all cell lines analyzed (35 genes). Right panel shows the full sensitivity analysis when the

fold change threshold is varied from 1.3 to 1.9. Details of the analysis can be found in the Methods section.

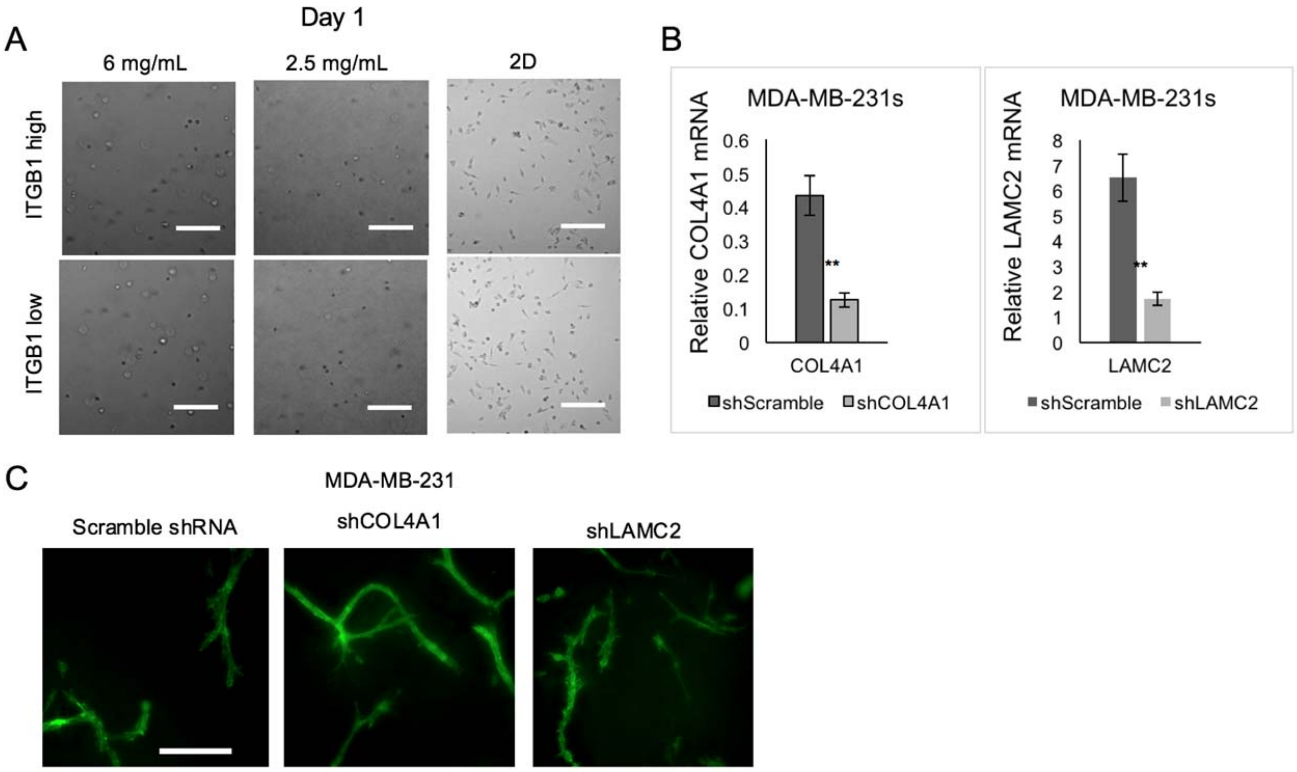

Supplementary Figure 3. A. ITGB1 sorted MDA-MB-231 cells at day 1 of embedding in high density and low density collagen matrices and plated on tissue culture plastic (2D). Scale bar 200 μm. **B.** RT-qPCR validation of shRNA mediated knock down of LAMC2 and COL4A1 **C.** Representative images of MDA-MB-231 cells expressing shRNA constructs against a scramble sequence, COL4A1, or LAMC2 after 7 days of culture in high density collagen Scale bar 200μm. N=3 biological replicates for all experiments unless otherwise noted. Statistical significance was determined by Wilcoxon rank sum test and is indicated as *, **, *** for p≤0.05, p≤0.01, p≤0001 respectively.

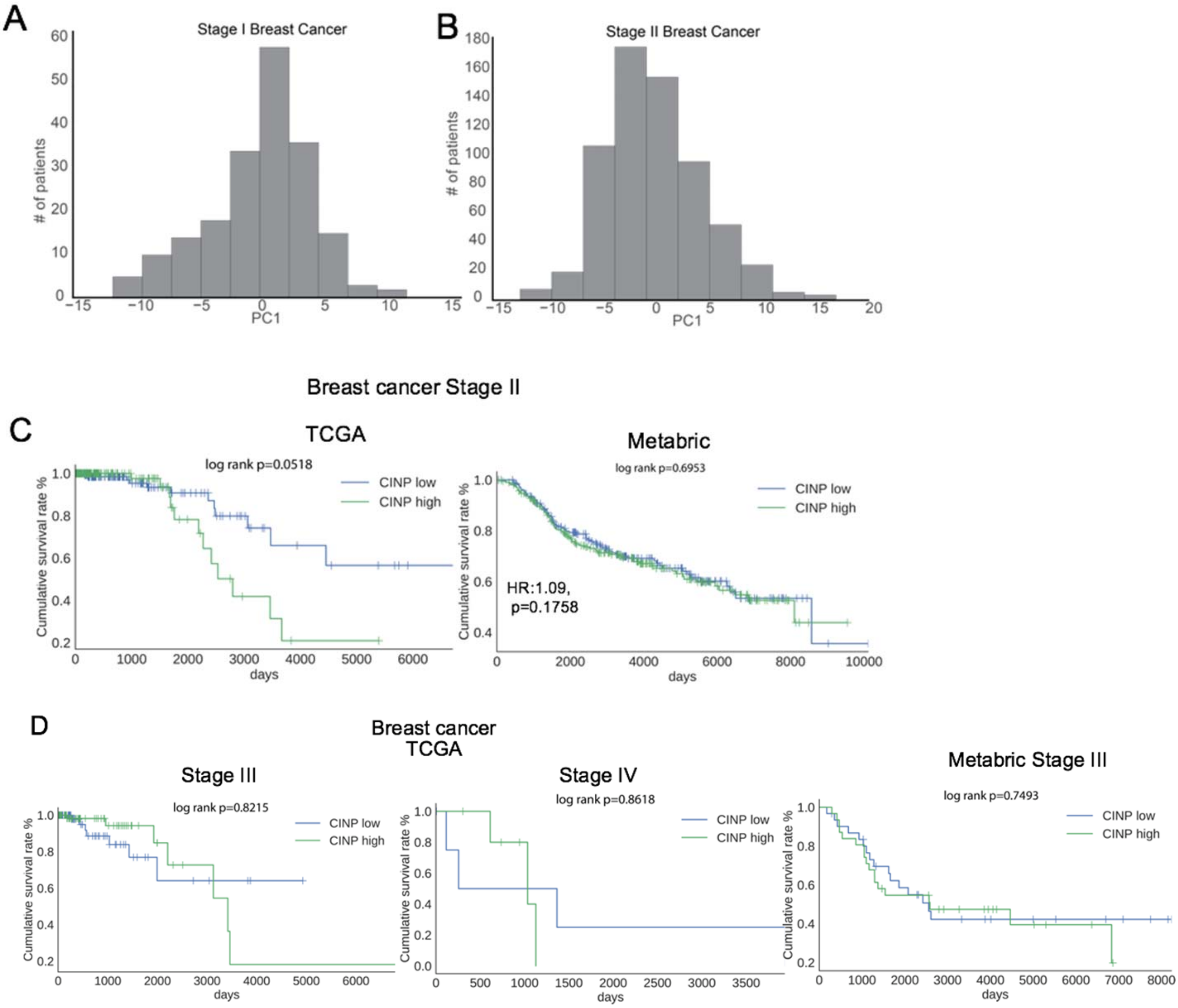

Supplementary Figure 4. A. Loadings of the first principal component (PC1) in stage I breast cancer patients of the 70 CINP associated genes identified in this study (Figure 2). **B.** Loadings of the first principal component (PC1) in stage II breast cancer patients of the 70 CINP associated genes identified in this study (Figure 2). **C.** Kaplan Meier survival analysis of stage II breast cancer patients in TCGA (left) and Metabric (right) databases when the PC1 loadings were used as an expression metagene. **D.** Kaplan Meier plots showing survival prediction by the CINP gene signature in Stage III and Stage IV breast cancer from TCGA data and stage III from metabric.

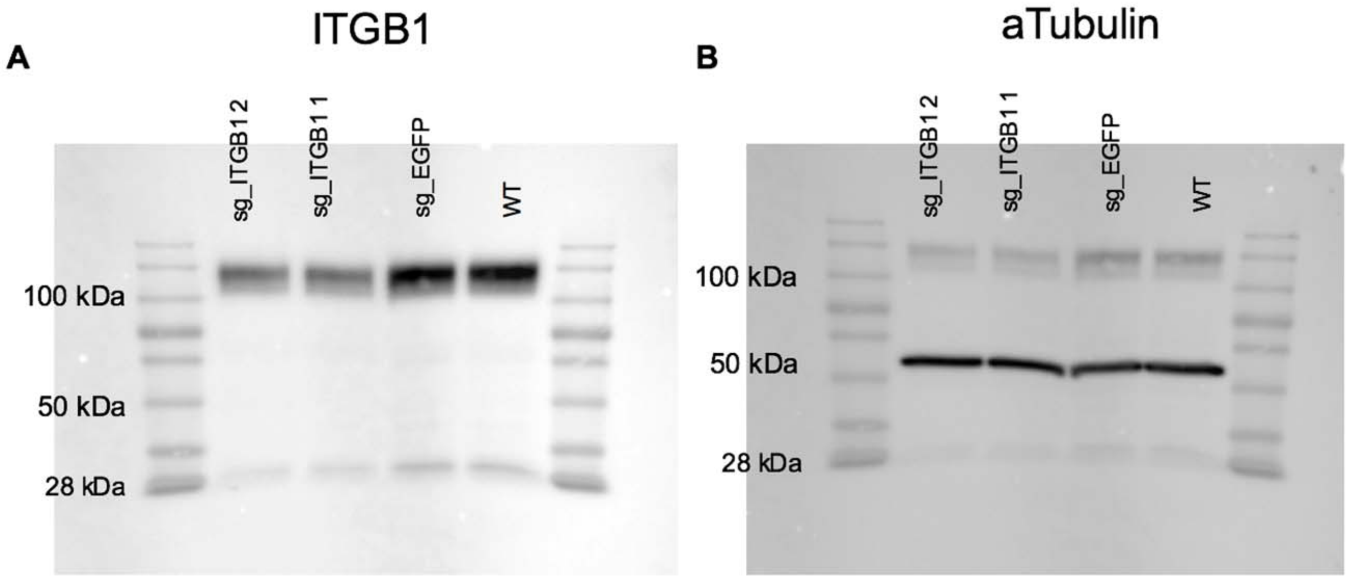

Supplementary Figure 5. Uncropped Western blots from Figure 4A. A. Integrin B1 Western blot. B. Alpha tubulin western blot.

Supplementary Table 1. Sensitivity analysis of GO enrichment

fcThreshold	geneList	Description	# genes in set	expectation	fold_enrich
1.3	70 Genes	blood vessel development	16	2.890	5.536
		regulation of cell migration	13	2.356	5.518
	35 Genes	cell differentiation	28	13.647	2.052
		regulation of smooth muscle cell migration	3	0.164	18.333
1.4	70 Genes	blood vessel development	12	1.630	7.361
		regulation of cell migration	12	1.329	9.030
	35 Genes	cell differentiation	19	7.696	2.469
		regulation of smooth muscle cell migration	3	0.092	32.511
1.5	70 Genes	blood vessel development	9	0.982	9.167
		regulation of cell migration	10	0.800	12.496
	35 Genes	cell differentiation	12	3.265	3.676
		regulation of smooth muscle cell migration	3	0.039	76.639
1.6	70 Genes	blood vessel development	8	0.667	11.997
		regulation of cell migration	8	0.544	14.718
	35 Genes	cell differentiation	6	1.982	3.027
		regulation of smooth muscle cell migration	1	0.024	42.076
1.7	70 Genes	blood vessel development	7	0.482	14.534
		regulation of cell migration	7	0.393	17.832
	35 Genes	cell differentiation	3	1.283	2.339
		regulation of smooth muscle cell migration	1	0.015	65.027
1.8	70 Genes	blood vessel development	6	0.333	17.995
		regulation of cell migration	4	0.272	14.718
	35 Genes	cell differentiation	3	0.933	3.216
		regulation of smooth muscle cell migration	1	0.011	89.413
1.9	70 Genes	blood vessel development	6	0.278	21.594
		regulation of cell migration	3	0.226	13.246
	35 Genes	cell differentiation	2	0.816	2.450
		regulation of smooth muscle cell migration	1	0.010	102.186

Supplementary Table 2. Gene ontology enrichment analysis for the genes in the 70 gene list

GO Term	#genes in set	genes in set
**regulation of cell migration**	10	EDN1|JAG1|PODXL|TPM1|HMOX1|FURIN|LAMB1|RBPJ|THBS1|SMAD7
**regulation of developmental process**	16	EDN1|JAG1|LTBP4|HPS4|THBS1|SMAD7|SIPA1L1|COL4A2|ID2|HMOX1|ITGAV|HES1|VHL|EPHB2|SKIL|NKX3-1
**regulation of cellular component movement**	10	EDN1|JAG1|PODXL|TPM1|HMOX1|FURIN|LAMB1|RBPJ|THBS1|SMAD7
**regulation of locomotion**	10	EDN1|JAG1|PODXL|TPM1|HMOX1|FURIN|LAMB1|RBPJ|THBS1|SMAD7
**anatomical structure development**	27	TAGLN|NLGN2|LAMC2|RBPJ|THBS1|SYNE1|LFNG|SIPA1L1|PODXL|HMOX1|ITGAV|HES1|IGF2BP3|VHL|EPHB2|SKIL|NKX3-1|EDN1|JAG1|TPM1|NAV1|LAMB1|SMAD7|COL5A1|COL4A1|ID2|KCTD11
**regulation of multicellular organismal process**	17	EDN1|NLGN2|JAG1|TPM1|FURIN|THBS1|SMAD7|SIPA1L1|COL4A2|ID2|BHLHE40|HMOX1|HES1|IGF2BP3|EPHB2|SKIL|NKX3-1
**system development**	25	TAGLN|NLGN2|LAMC2|RBPJ|THBS1|LFNG|SIPA1L1|PODXL|HMOX1|ITGAV|HES1|VHL|EPHB2|SKIL|NKX3-1|EDN1|JAG1|TPM1|NAV1|LAMB1|SMAD7|COL5A1|COL4A1|ID2|KCTD11
**developmental process**	29	TAGLN|NLGN2|LTBP4|LAMC2|FURIN|RBPJ|THBS1|SYNE1|LFNG|SIPA1L1|PODXL|HMOX1|ITGAV|HES1|IGF2BP3|VHL|EPHB2|SKIL|NKX3-1|EDN1|JAG1|TPM1|NAV1|LAMB1|SMAD7|COL5A1|COL4A1|ID2|KCTD11
**blood vessel development**	9	EDN1|JAG1|COL5A1|COL4A1|HMOX1|ITGAV|VHL|THBS1|SMAD7
**vasculature development**	9	EDN1|JAG1|COL5A1|COL4A1|HMOX1|ITGAV|VHL|THBS1|SMAD7
**cellular component organization**	25	NLGN2|LAMC2|RBPJ|THBS1|SYNE1|MRC2|SIPA1L1|ABLIM3|HMOX1|ITGAV|HES1|VHL|EPHB2|SKIL|TPM1|HPS4|NAV1|LAMB1|H2BFS|SMAD7|DAAM1|COL4A2|COL5A1|LPCAT2|TGFBI
**anatomical structure formation involved in morphogenesis**	10	EDN1|JAG1|COL4A1|PODXL|TPM1|HMOX1|VHL|THBS1|SKIL|NKX3-1
**anatomical structure morphogenesis**	17	EDN1|JAG1|TPM1|LAMB1|THBS1|SMAD7|LFNG|COL5A1|COL4A1|PODXL|HMOX1|HES1|IGF2BP3|VHL|EPHB2|SKIL|NKX3-1
**regulation of transforming growth factor beta receptor signaling pathway**	5	LTBP4|FURIN|THBS1|SKIL|SMAD7
**organ development**	20	EDN1|TAGLN|JAG1|TPM1|LAMC2|LAMB1|THBS1|SMAD7|LFNG|COL5A1|COL4A1|PODXL|ID2|HMOX1|ITGAV|HES1|VHL|EPHB2|SKIL|NKX3-1
**multicellular organismal development**	26	TAGLN|NLGN2|LTBP4|LAMC2|RBPJ|THBS1|LFNG|SIPA1L1|PODXL|HMOX1|ITGAV|HES1|VHL|EPHB2|SKIL|NKX3-1|EDN1|JAG1|TPM1|NAV1|LAMB1|SMAD7|COL5A1|COL4A1|ID2|KCTD11
**negative regulation of cellular process**	20	EDN1|JAG1|TPM1|AMIGO2|FURIN|RBPJ|THBS1|SMAD7|PODXL|ID2|BHLHE40|HMOX1|ITGAV|HES1|IGF2BP3|VHL|TGFBI|EPHB2|SKIL|NKX3-1
**negative regulation of biological process**	21	EDN1|JAG1|TPM1|AMIGO2|FURIN|RBPJ|THBS1|SMAD7|COL4A2|PODXL|ID2|BHLHE40|HMOX1|ITGAV|HES1|IGF2BP3|VHL|TGFBI|EPHB2|SKIL|NKX3-1
**regulation of cell differentiation**	11	EDN1|SIPA1L1|JAG1|ID2|LTBP4|ITGAV|HES1|VHL|EPHB2|SKIL|SMAD7

Supplementary Table 3. Gene ontology enrichment analysis for the genes in the 35 gene list

GO Term	#genes in set	genes in set
**regulation of smooth muscle cell migration**	3	ITGA2|SERPINE1|TRIB1
**cell differentiation**	12	SEMA7A|UHRF2|CHST11|GADD45B|ITGA2|SPHK1|FN1|FZD8|ULK1|JARID2|FSTL3|IGF1R
**cellular developmental process**	12	SEMA7A|UHRF2|CHST11|GADD45B|ITGA2|SPHK1|FN1|FZD8|ULK1|JARID2|FSTL3|IGF1R
**regulation of cell migration**	5	ITGA2|SPHK1|SERPINE1|TRIB1|IGF1R
**developmental growth**	4	CHST11|SERPINE1|PLAUR|ULK1
**negative regulation of smooth muscle cell migration**	2	SERPINE1|TRIB1
**regulation of cellular component movement**	5	ITGA2|SPHK1|SERPINE1|TRIB1|IGF1R
**regulation of locomotion**	5	ITGA2|SPHK1|SERPINE1|TRIB1|IGF1R
**positive regulation of cell migration**	4	ITGA2|SPHK1|SERPINE1|IGF1R
**positive regulation of cellular component movement**	4	ITGA2|SPHK1|SERPINE1|IGF1R
**positive regulation of locomotion**	4	ITGA2|SPHK1|SERPINE1|IGF1R
**regulation of protein metabolic process**	7	NDUFA13|ITGA2|SPHK1|SERPINE1|PLAUR|JARID2|TRIB1
**regulation of cellular component organization**	6	ITGA2|SPHK1|SERPINE1|FN1|ULK1|JARID2
**positive regulation of smooth muscle contraction**	2	ITGA2|SPHK1
**growth**	4	CHST11|SERPINE1|PLAUR|ULK1
**positive regulation of cellular component organization**	4	ITGA2|SPHK1|SERPINE1|JARID2
**positive regulation of muscle contraction**	2	ITGA2|SPHK1
**regulation of cell proliferation**	7	CHST11|ITGA2|SPHK1|SERPINE1|JARID2|TRIB1|IGF1R

Supplementary table 4. TCGA analysis not significant effect cox p >0.05

Cancer type	Patient count	Death Observed	HR	Cox p
**BRCA**	1131	104	1.1116	0.3268
**UCEC**	555	45	1.2440	0.1955
**HNSC**	518	167	1.1604	0.0748
**PRAD**	505	8	1.3992	0.4090
**THCA**	504	14	1.0754	0.8104
**COAD**	499	59	0.8250	0.1646
**LUSC**	489	154	1.0746	0.3913
**LIHC**	369	89	1.1355	0.2679
**OV**	337	185	1.0028	0.9721
**KIRP**	287	32	1.2456	0.2371
**STAD**	279	77	1.2858	0.0571
**SARC**	257	75	0.9090	0.4110
**PCPG**	179	6	0.8633	0.7016
**READ**	165	9	0.5978	0.3112
**GBM**	156	53	1.1312	0.2311
**TGCT**	133	3	0.9231	0.9001
**THYM**	120	6	1.0127	0.9496
**ESCA**	119	57	0.7878	0.6258
**SKCM**	93	10	1.6624	0.2016
**UVM**	80	13	1.4671	0.1305
**UCS**	57	25	0.8913	0.5450
**DLBC**	47	5	0.9887	0.9806
**CHOL**	36	16	1.0232	0.9343

Supplementary Table 4. Cancer types for which there is data available in TCGA but no significant differences between high and low CINP groups were detected. Table shows number of patients available, number of deaths reported, hazard ratio and cox model p value. See methods for analysis details.
